# A new plant guanosine cyclase ZjGC found from jujube regulates growth and development via endogenous hormones

**DOI:** 10.3389/fpls.2025.1633496

**Published:** 2025-09-04

**Authors:** Xin Zhao, Haonan Cao, Yao Liu, Junyan Mai, Fangyuan Yang, Zhiguo Liu, Mengjun Liu

**Affiliations:** ^1^ College of Horticulture, Hebei Agricultural University, Baoding, China; ^2^ Agriculture and Rural Bureau of Luanzhou, Tangshan, China; ^3^ Research Center of Chinese Jujube, Hebei Agricultural University, Baoding, China

**Keywords:** guanosine cyclase, jujube, ABA, GA, seed germination, flowering

## Abstract

Cyclic guanosine monophosphate (cGMP) is an important second messenger involved in many physiological processes. Guanylate cyclase (GC), the key synthetase of cGMP, has been identified in many microorganisms and mammals, but very few in plants for their poor cGMP level. The biological functions of GC and endogenous cGMP in plant remains largely unknown. Here, we found a new plant GC, *ZjGC*, from jujube (*Ziziphus jujuba* Mill.) rich in cGMP via genome-wide identification, and its function catalyzing cGMP synthesis was confirmed by *in vitro* enzymatic property analysis, transient overexpression in jujube fruit *in vivo*, and generation of transgenic plants of Micro-Tom tomato. Overexpressing *ZjGC* in tomato showed that *ZjGC* has vital biological functions of promoting seed germination, restraining plant height growth, shortening juvenile period, accelerating fruit development, increasing seed number, and decreasing the size of fruit and seed by increasing endogenous cGMP content with significant increase of GA3 and moderate decrease of IAA and ABA. A feedback regulation mechanism of cGMP to hormones GA3 and ABA was firstly discovered in plant. Adding cGMP to jujube calli significantly increased GA3 content, promoted calli growth and differentiation. This research lays a solid foundation for further study and utilization of GC and cGMP as well as the research on phytohormone signaling in plants.

## Introduction

cGMP (cyclic guanosine monophosphate) is a key intracellular second messenger (hormone as the first messenger), has been proved to participate in a wide range of cell metabolism regulation and functions (Newton et al., 1999; Talke et al., 2003). In plants, cGMP plays a critical regulatory role in many physiological processes, signal transduction, and stress response (Zhai et al., 2024). It was found that cGMP is involved in auxin-induced adventitious root formation (Palme and Redhead, 1997), chloroplast development (Bowler et al., 1994), pollen germination and pollen tube growth (Prado et al., 2004), as well as anthocyanin and flavonoid synthesis (Suita et al., 2009). Additionally, cGMP also acts as a signaling molecule in plant defense mechanisms, such as stomata regulation (Dubovskaya et al., 2011) and plant tolerance to stress (Ederli et al., 2014). Furthermore, cGMP is required for gibberellic acid-induced seed germination (Wu et al., 2013), and works in conjunction with nitric oxide to regulate root growth and bending toward the ground (Li and Xue, 2010; Nan, 2016a; Qi et al., 2023), along with photoperiod-controlled flowering (Jia et al., 2018). In addition, cGMP participates in plant immune response, which can induce miRNA expression (Lin et al., 2012) and respond to pathogens rapidly (Duszyn et al., 2022).

Guanosine cyclase (GC) is the key enzyme that catalyzes GTP to 3’,5’-cGMP (3’,5’-cyclic guanosine monophosphate) (Denninger and Marletta, 1999). In fact, identifying GC in higher plants is a much more difficult task because the content of cGMP in plants is very low and BLAST search cannot identify any matching annotated GC from higher plants. This suggests that higher plants have evolved unique GC molecules, in which only the catalytic center may exhibit some degree of conservation (Świeżawska et al., 2017). Therefore, only a few GC have been identified in the plant kingdom (Ludidi and Chris, 2003; Horst et al., 2019; Rahman et al., 2020).

However, the above reports are all based on the studies of exogenous cGMP or phytohormones. It is still unclear how endogenous cGMP works in plants and whether cGMP can reversely regulate phytohormones? Moreover, all the previous studies on the biological functions of exogenous cGMP focused on a single or few aspects, there is still a lack of systematic understanding on cGMP, especially endogenous cGMP, regulating the growth and development of higher plants.

In this study, we successfully identified and characterized a plant GC (ZjGC) for the first time from Chinese jujube (*Ziziphus jujuba* Mill.), a fruit tree with super high cGMP content in mature fruit. We then overexpressed the *ZjGC* gene in model plant tomato and made a systematic study of its crucial biological functions and the underlying mechanisms of endogenous cGMP in plants. This study lays a good foundation for further research of plant GC and cGMP and sheds light on the feedback regulation of cGMP to the primary messenger hormone in the control of growth and development in plants.

## Result

### Genome-wide identification of candidate *GC* gene in jujube

We used the *GC* gene sequence of *Arabidopsis thaliana* (*AtGC1*) as the query condition to search the jujube genome database. Only one candidate *GC* fragment cDNA sequence (828 base pairs) was determined by homologous cloning from the total RNA of ‘Dongzao’ jujube, and sequenced after transformation into *Escherichia coli* DH5α. The predicted CDS has 100% identity with the above 828 bp sequence. Finally, the CDS was isolated and sequenced by PCR and named as *ZjGC*.

### Comparison and evolutionary analysis of the jujube ZjGC protein with known GCs

**Figure 1:**
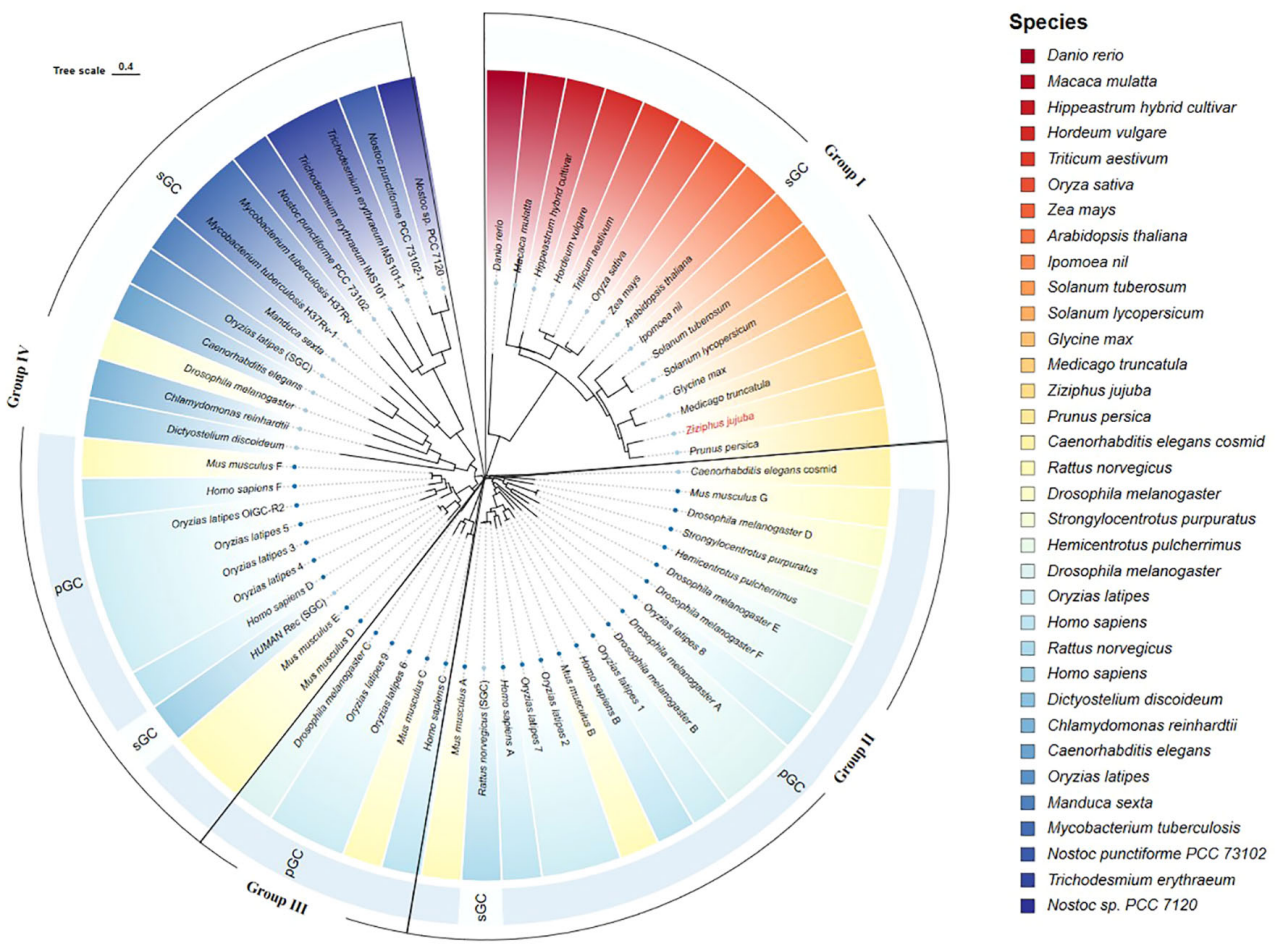
Phylogenetic analysis of the *ZjGC* protein with other published GCs. The GCs from different species were distinguished with different colors, among them, two GC types (sGC and PGC) were represented by two lighter blue colors, which together formed the outer ring of the evolutionary tree, and four groups were also marked with different shapes.

The ZjGC protein conserved domains was used to search in NCBI and found that ZjGC belongs to Guanylate cyc 2 superfamily (GC-2) which belongs to soluble GC (sGC). We used the MEGA software to compare the amino acid sequence of the ZjGC with the known GCs of animals and plants. From **Figure 1**, we can see that GCs are mainly divided into 4 main groups: group I is mainly composed of plant GCs, group II and group III are mainly composed of animal GCs, group IV includes GCs from animals and some algae and insects. Of them, the jujube GC (ZjGC) is closest to peach on the branch of group I.

### Transient overexpression of *ZjGC* into jujube fruit *in vivo*

**Figure 2:**
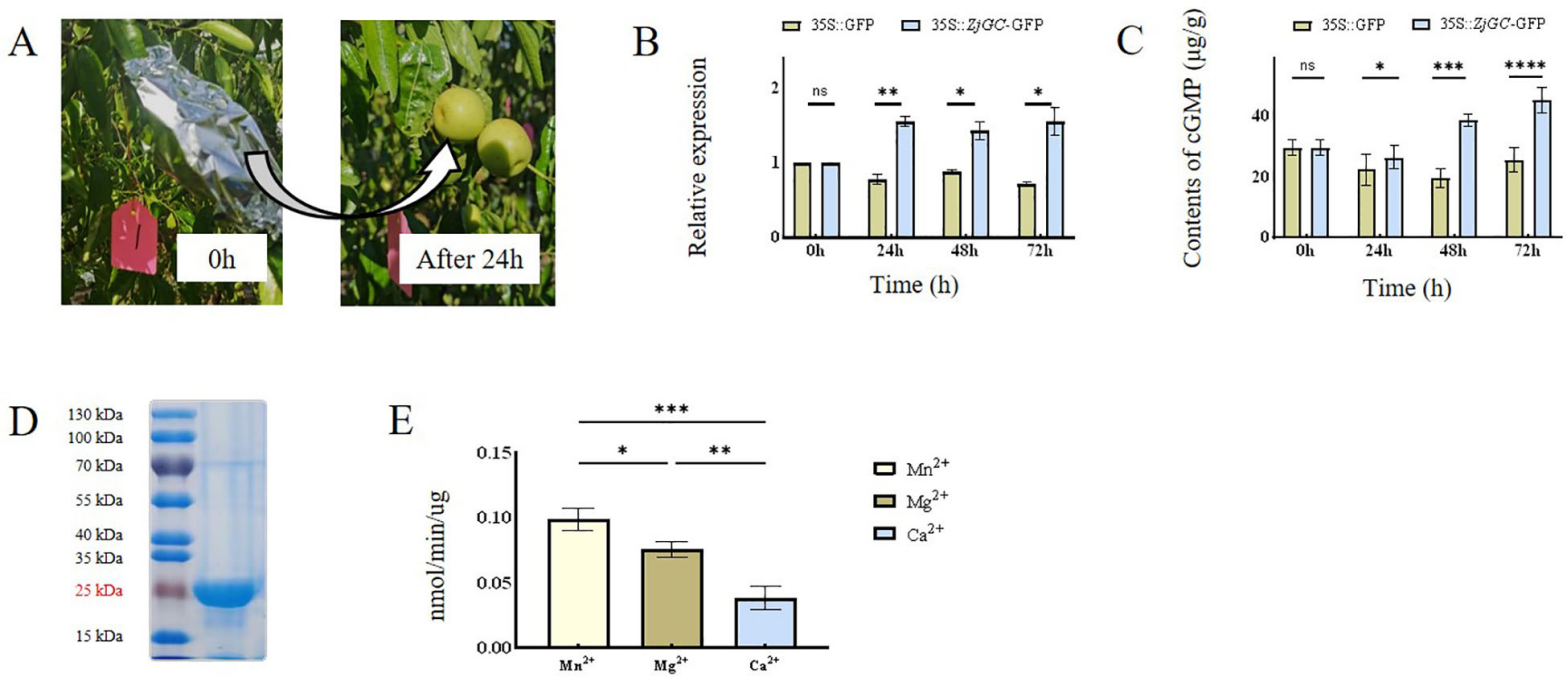
The catalytic function of *ZjGC* gene. **(A)** Comparison of fruits before and after 24-hour dark treatment following *Agrobacterium* injection. **(B)** The expression level of *ZjGC* in transient overexpression Chinese jujube fruits. **(C)** The cGMP contents in transient overexpression Chinese jujube fruits. **(D)** The SDS protein gel stained and the activity of ZjGC. **(E)** Activity assay of ZjGC. Significant differences were based on Student's t-test: P<0.05. Error bars indicate ±SD (n = 3).

To elucidate whether the *ZjGC* regulates cGMP synthesis, *in vivo* white-mature stage jujube fruits in three independent trees were used for transient overexpression analysis. After 24 h of treatment (**Figure 2A**), as shown in **Figure 2B**, the expression of *ZjGC* was increased in the transgenic fruits, and the contents of cGMP were significantly higher than those of the negative control (**Figure 2C**), proving that the ZjGC can catalyze the synthesis cGMP.

### *In vitro* confirmation of the enzymatic properties of ZjGC

We cloned, expressed, and purified the ZjGC protein. A main band with an apparent molecular mass of 25 kDa was recognized from SDS-poly-acrylamide gel electrophoresis (**Figure 2D**). The size is consistent with the predicted theoretical value. Its enzyme activity was then determined by detecting the cGMP content of the reaction product, and the calibration curve covered the range of cGMP measurements in the experiments. Meanwhile, we figured out that in the presence of divalent cation, Mn^2+^ was most helpful for ZjGC activity (**Figure 2E**), and could assist protein to approach its highest activity of 0.098 nmol/min/μg. Mg²^+^ as a cofactor, could also provide effective support, the ZjGC activity reached 0.076 nm/min/μg. Compared to Mn^2+^ and Mg^2+^, the enzyme activity with Ca²^+^ as a cofactor was the lowest. The results showed that recombinant ZjGC can catalyze the synthesis of cGMP from GTP.

### Generation of *ZjGC* overexpression transgenic plants of Micro-Tom tomato

To further investigate the expression regulation mode and the biological functions of *ZjGC*, we generated *ZjGC* overexpression transgenic tomato plants using 35S::*ZjGC*-GFP and 35S::GFP. After the *Agrobacterium*-mediated genetic transformation, the positive tissue culture seedlings were verified by RT-PCR, indicating that the expression vector constructed had been integrated into the tomato genome. The transgenic plant seedlings with the highest *GC* expression level and healthy physiological state were selected for subsequent experiments and analyses.

### Overexpression of *ZjGC* accelerates seed germination

**Figure 3:**
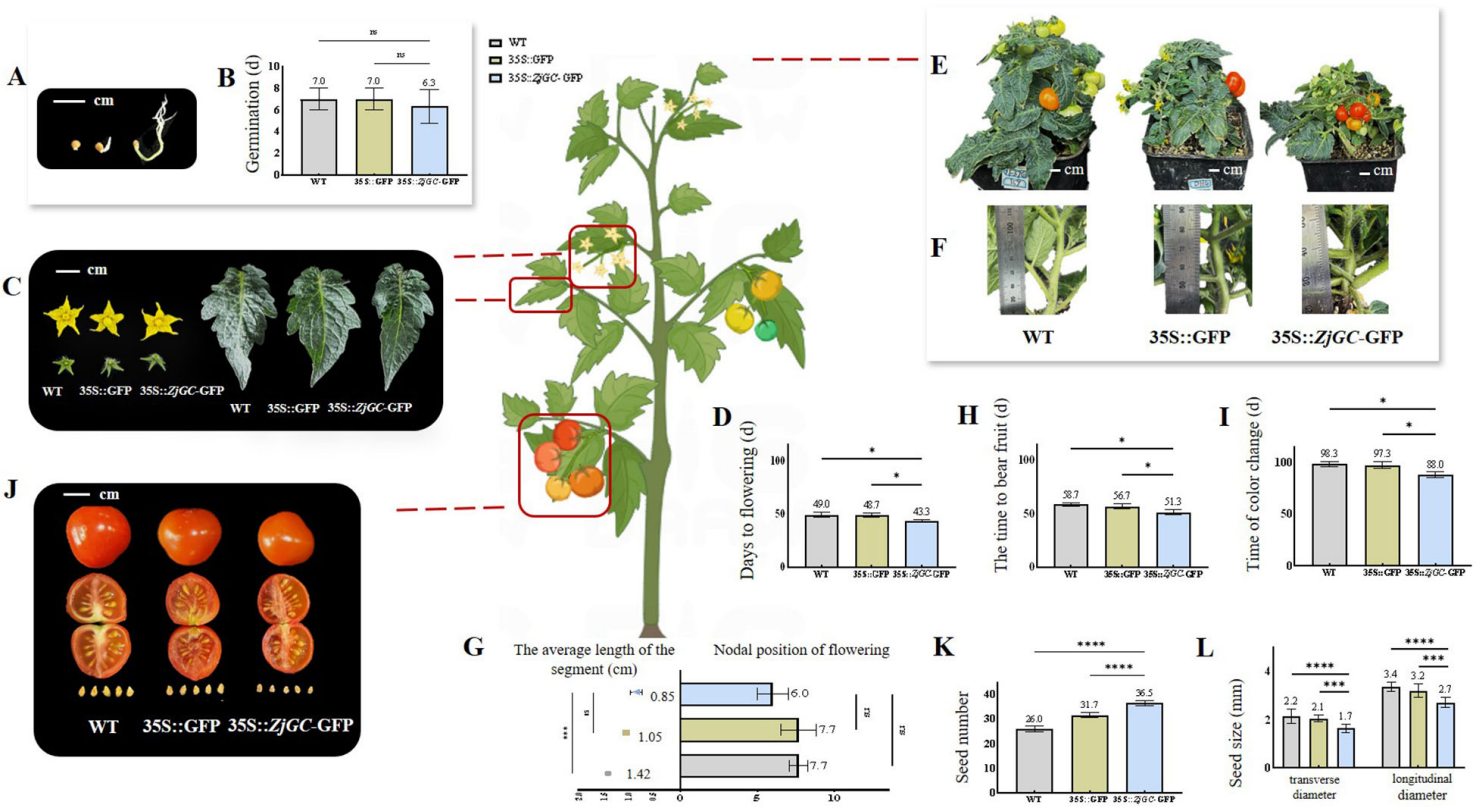
The biological effects of *ZjGC* overexpressing in tomato. **(A)** Vector plot of seed germination. **(B)** The germination of seed. **(C)** Representative images of flowers and leaves. **(D)** The days needed from sowing seed to flowering. **(E)** Representative images showing plant height. **(F)** Representative images showing the average node length of plants. **(G)** The flowering node position and average node length of plants. **(H)** The days needed to bear fruit. **(I)** The days needed for color change of fruit. Significant differences were based on Student's t-test: P < 0.05. Data are mean ± SD (n = 9). **(J)** Representative images of mature fruits. **(K)** The seed numbers. **(L)** The seed size. Significant differences were based on Student's t-test: P<0.05. Error bars indicate ± SD of 40 seeds.

From **Figures 3A, B** we can see that all the *ZjGC* overexpressing transgenic lines showed much earlier seed germination than wild type (WT), suggesting that the cGMP may function as a positive regulator for seed germination.

### Overexpression of *ZjGC* regulates phase change and fruit development

The flowering time of *ZjGC* overexpression transgenic lines was significantly earlier, and the size of the flowers and leaves did not change (**Figure 3C**). The average flowering time of WT and 35S::GFP was about 49 d after seed sowing, but 35S::*ZjGC-GFP* was only 43.3 d after seed sowing, nearly 6 days earlier than that of the control (**Figure 3D**). In addition, the transgenic 35S::*ZjGC*-GFP tomato plants were significantly shorter than the WT plants (**Figures 3E, F**), so we measured the flowering node position and inter-node spacing of all plants. As shown in **Figure 3G**, the flowering node position of 35S::*ZjGC*-GFP tomato plants was 6, which was 1.7 nodes less than that of the control. The average node length of 35S::*ZjGC*-GFP was also the shortest, which was only 0.85 cm, about 0.57 cm shorter than WT. These results suggest that the *ZjGC* gene positively regulates plant phase change and flowering.

The average time for WT and 35S::GFP to bear fruit after seed sowing were 58.7 d and 56.7 d, respectively, but only 51.3 d for 35S::*ZjGC*-GFP, 5–7 d earlier (**Figure 3H**). The average time from seed sowing to fruit coloring of WT and 35S::GFP was 98.3 d and 97.3 d (**Figure 3I**), while 35S::*ZjGC*-GFP was only 88 d, 9–10 days earlier. From fruit setting to fruit color change, the development period of WT fruit was 39.6 d, 35S::GFP was 40.6 d, and *ZjGC* overexpression transgenic fruit was only 36.7 d. The results showed that the *ZjGC* gene has the function of accelerating fruit growth and development. Also, we can see that *ZjGC* affects the fruit size and appearance (**Figure 3J**), the *ZjGC* overexpression transgenic fruit showed significant smaller fruit (**Table 1**), thinner mesocarp and lighter skin color in mature fruit.

**Table 1:** The fruit weight and appearance of *ZjGC* overexpression transgenic lines and WT plants.

Tomato Lines	Single fruit weight (g)	Longitudinal diameter (mm)	Transverse diameter (mm)	L*(D65)	a*(D65)	b*(D65)
WT	4.89 ± 1.00a	21.76 ± 2.10a	20.23 ± 1.59a	25.84 ± 3.81	18.05 ± 4.04	14.73 ± 6.37
35S::GFP	4.65 ± 0.61a	21.31 ± 0.76a	19.54 ± 1.87a	24.65 ± 2.96	21.1 ± 2.86	17.78 ± 3.76
35S::*ZjGC*-GFP	3.26 ± 0.22b	18.50 ± 0.91b	17.29 ± 0.29a	22.99 ± 2.4	20.23 ± 3.26	15.18 ± 5.27

Fruit color was measured in the CIE L*a*b* color space. L*: Lightness; a*: Green-Red Axis; b*: Blue-Yellow Axis. Significant differences were based on Student’s t-test: P < 0.05. Error bars indicate ± SD of 20 fruits.

### Overexpression of *ZjGC* promotes seed formation

Besides, it was found that the number of seeds in the transgenic tomato fruit was significantly higher than that in the WT. The average number of seed in the WT and 35S:: GFP tomato fruits was 26 and 31.7, while the 35S:: *ZjGC*-GFP tomato fruit was 36.5, increasing over 40% compared to the WT (**Figure 3K**). Therefore, the results demonstrated that the *ZjGC* has a biological function in promoting seed formation. In addition, *ZjGC* also affects seed size (**Figure 3L**). The transverse and longitudinal diameters of WT tomato seeds were 2.2 mm and 3.4 mm, respectively, while those of 35S::GFP seeds were 2.1 mm and 3.2 mm. In contrast, the seeds of the transgenic tomatoes were significantly smaller than the former two, with transverse and longitudinal diameters of 1.7 mm and 2.7 mm, respectively.

### Effects of exogenous cGMP on jujube calli development

On the 10th day after treatment, the medium added with cGMP of 200 and 300 μM had the greatest effect on the calli growth rate, both of them were all reached 68% (**Table 2**). On the 20th day, the best one was 100μM cGMP, with calli growth rate reaching 145%, 1.79 times of the control. The results showed that cGMP could promote the growth of calli.

**Table 2:** The effects of exogenous cGMP on jujube callus growth rate.

Reagent	Concentration (μM)	10d	20d
	0	52% b	81% bc
cGMP	50	59% ab	48% d
	100	32% c	145% a
	200	68% a	72% c
	300	68% a	90% b

The calli growth rate = (M - M0)/M0 × 100% (M is the calli mass of each sampling; M0 is the initial calli mass). Significant differences were based on Student’s t-test: P < 0.05. Error bars indicate ± SD (n = 3).

**Figure 4:**
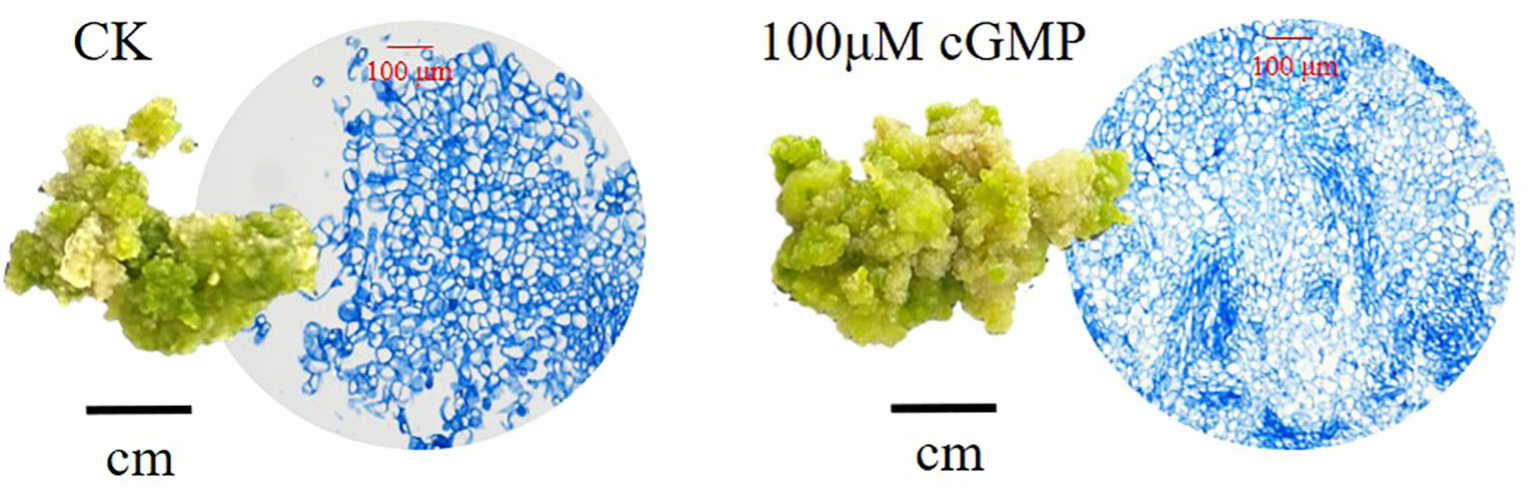
Comparison of calli appearance and cell size on the 20th day. On the left is calli cultured without exogenous cGMP (CK), while on the right is calli cultured with 100 μM exogenous cGMP.

As shown in **Figure 4**, exogenous cGMP could cause morphological changes of jujube calli. For example, on the 20th day after treated with 100μM cGMP, jujube calli were tight and rigid with many small cells, presenting a type I callus shape that is easy to differentiate into buds.

### Feedback regulations of cGMP on endogenous hormones

**Figure 5:**
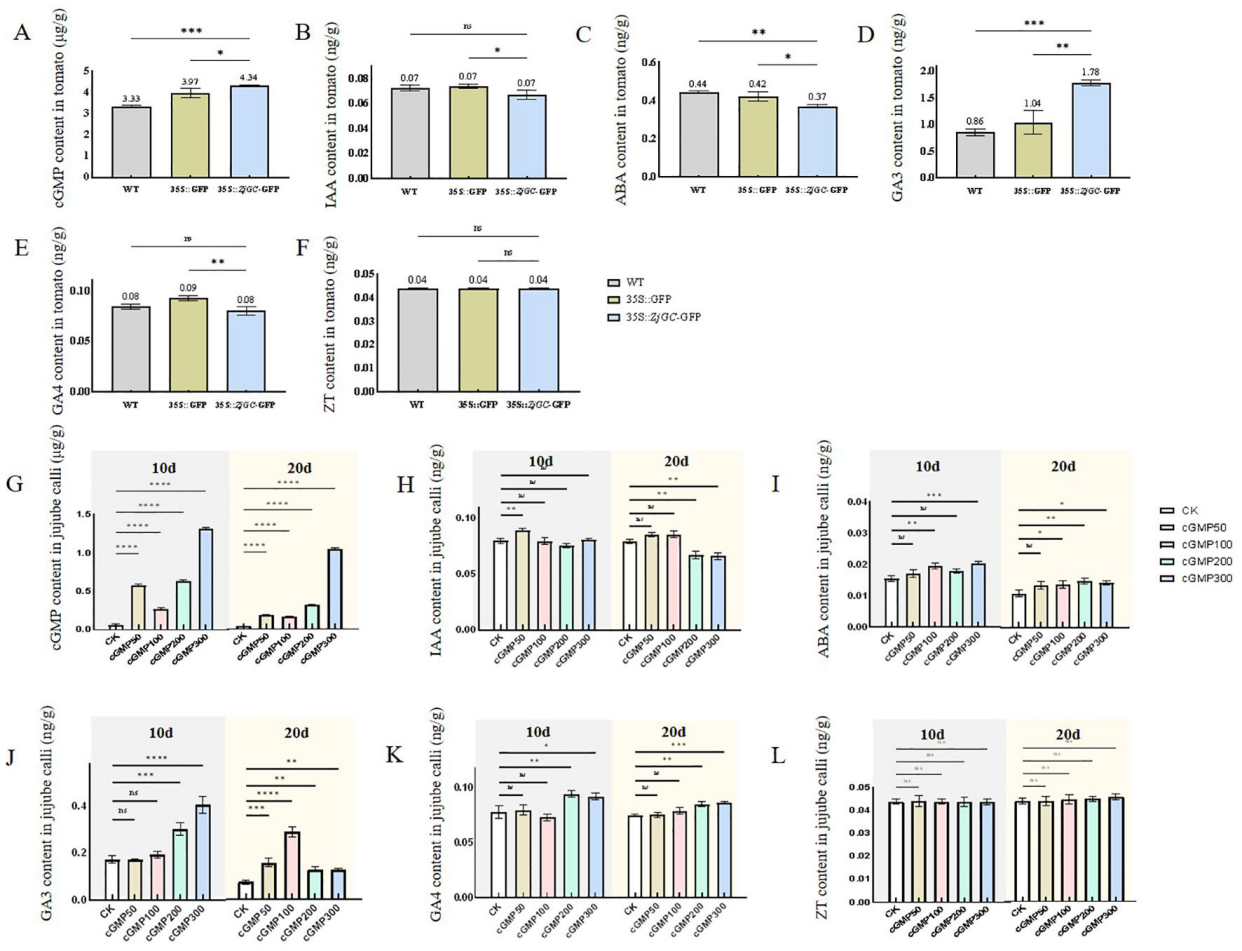
Changes in cGMP and hormone contents of *ZjGC* overexpression tomatoes and jujube calli. **(A)** The cGMP content in tomato lines. **(B)** The IAA content in tomato lines. **(C)** The ABA content in tomato lines. **(D)** The GA3 content in tomato lines. **(E)** The GA4 content in tomato lines. **(F)** The ZT content in tomato lines. **(G)** The cGMP content in jujube calli. **(H)** The IAA content in jujube calli. **(I)** The ABA content in jujube calli. **(J)** The GA3 content in jujube calli. **(K)** The GA4 content in jujube calli. **(L)** The ZT content in jujube calli. Significant differences were based on Student's t-test: P<0.05. Error bars indicate ± SD (n = 3).

It can be seen from **Figure 5** that the content of cGMP in the overexpressing transgenic tomato plants was extremely higher than that of the control, indicating that *ZjGC* can catalyze the generation of cGMP in transgenic tomato plants. The hormone content analysis showed that IAA content and ABA content in 35S::*ZjGC*-GFP overexpressing transgenic fruits were significantly lower than those in WT by 0.005 ng/g (7.39%) and 0.07 ng/g (16.21%), respectively. GA4 content was also lower than WT. However, GA3 content in overexpressed tomato fruits was 0.92 ng/g (2.08-fold) higher than that in WT and 0.74 ng/g (1.71-fold) higher than that in 35S::GFP.

From **Figure 5**, we can see that exogenous cGMP can cause a significant increase in cGMP content in jujube calli. However, on the day 20, IAA contents in calli treated with cGMP of 200μM and 300μM were significantly decreased by 15.15% and 16.41%, respectively. Exogenous cGMP also increased the content of ABA on the 10th day, with 100μM and 300μM more efficient, increasing 26.05% and 32.27%, respectively. The highest effect of cGMP was found in GA3. On the 10th day, the content of GA3 in the calli treated with 200μM and 300μM cGMP increased by 74.68% and 134.22% compared with CK, respectively. On the 20th day, the content of GA3 in the jujube calli treated with 100μM cGMP even increased 293.87%. Furthermore, exogenous cGMP treatment at concentrations of 200μM and 300μM on the 10th day increased the content of GA4 in jujube calli by 21.13% and 17.93%, respectively. In addition, we found that exogenous cGMP did not affect ZT content in jujube calli on the 10th day, and only a slight increase on the 20th day.

## Discussion

The identification of GC in higher plants is a complicated and arduous work due to the specific sequence of GC in higher plants is evolutionarily different from animals and other microorganism (Mulaudzi et al., 2011), and cGMP has a very low content in plants. Since Ludidi and Chris (2003) first identified GC in *Arabidopsis* based on the conserved and functionally assigned amino acids in the catalytic centers of annotated GC, new explorations have been initiated into GC in higher plants, site directed mutagenesis has led to the discovery of more GC (Rahman et al., 2020; Wong and Gehring, 2013). Even so, GC has been identified and confirmed in only a few plant species like *Brachypodium distachyon* (Duszyn et al., 2021), *Chlamydomonas reinhardtii* (Horst et al., 2019) and tomato (Rahman et al., 2020). On the other hand, the studies on the biological function of GC protein in higher plants are even less. In *Arabidopsis*, overexpressing-*AtGC1* and lacking-*AtGC1* did not exhibit biological variations (Nan, 2016b). Before our research, the biological functions of GC or endogenous cGMP in plants were still unclear.

The present research identified and confirmed the first *GC* gene in jujube, named *ZjGC*. A systematic observation and analysis of the biological functions of GC/cGMP in plant and the underlying mechanism were also made for the first time by over-expressing of *ZjGC* in jujube and model plant tomato. We found that the seed germination of *ZjGC*-overexpressed tomato was obviously advanced; *ZjGC* significantly promoted plant phase change process by reducing the node number needed for flowering and shortening fruit period; *ZjGC* can affect fruit size and appearance, the transgenic tomato showed significant smaller fruit, thinner mesocarp and lighter skin color in mature fruit; At the same time, the *ZjGC*-overexpressed tomato produced more and smaller seeds.

**Figure 6:**
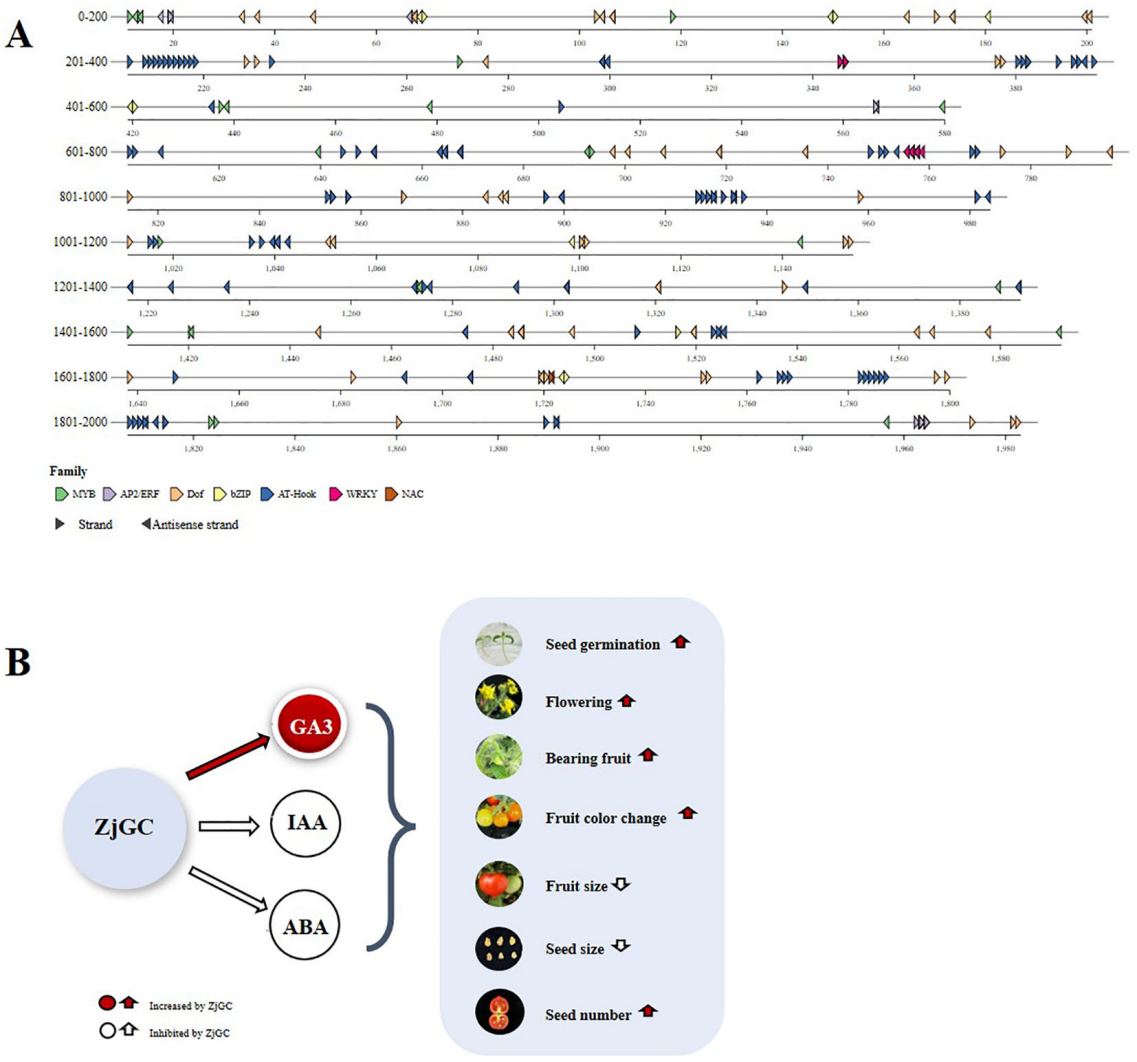
Predicted binding of *ZjGC* promoter to hormone-related transcription factors and the pathway of *ZjGC* gene regulating plant growth through hormones. **(A)** Distribution of transcription factor binding sites (TFBS) in the *ZjGC* promoter region (2-kb), and different colors represent specific TFBS bound to respective promoter segments. **(B)** Hypothesis figure of *ZjGC* regulating plant growth and development via influencing hormones. Red indicates positive regulatory effect, and white indicates negative regulatory effect.

Previous reports showed that hormones are the first messengers, while cGMP is the second messenger (Kwiatkowski et al., 2024), with the former regulating plant growth and development via the latter. Most previous tests (Teng et al., 2010; Nan et al., 2014) suggested that cGMP acts at the downstream of IAA, GA and ABA, and could not function without the upstream hormone. It was also pointed out that various hormones in plants can cause cGMP-dependent changes (Wu et al., 2013; Isner et al., 2012). cGMP mediates changes in the levels of various hormones and induces divalent ions within the plant, thus affecting the physiological processes of the plant in different ways (Shen, 2019). In fact, we used the bioinformatics analysis tool PlantPAN to analyze the 2000 bp promoter sequence upstream of the *ZjGC1* gene (**Figure 6A**), and found that the promoter sequence has several cis-acting elements associated with IAA, ABA, GA and JA (jasmonic acid) binding responses (MYB, AP2/ERF, Dof, bZIP, AT-hook, WRKY, and NAC). And the latest research also indicates that just like cAMP, the second messenger cGMP is closely related to the hormone IAA (Qi et al., 2023; Chen et al., 2025). And through the above promoter analysis, this undoubtedly proves that there is some feedback regulation between cGMP and hormones.

Our present research found an interesting phenomenon: endogenous cGMP can change the contents of plant hormones and regulate various biological processes. In fact, our results showed that, overexpressing *ZjGC* in tomato led to a significant increase of the cGMP content in fruit, together with a synergistic significant increase of GA3 content and a significant decrease of the contents of GA4, IAA and ABA, resulting in shortened juvenile phase (early flowering) and fruit development period, small fruit, more and small seeds. Basing on above mentioned, we assumed a hypothesis of cGMP regulating plant growth and development via influencing hormones (**Figure 6B**). According to previous research, we hypothesize that cGMP works together with GA3 and GA4 to promote floral transition by increasing GA3 levels (Castro-Camba et al., 2022) and decreasing GA4 levels (Huang et al., 2019). On the other hand, cGMP can negatively regulate fruit development by inhibiting IAA, which limits fruit expansion and seed growth (Liu et al., 2020). Moreover, cGMP may promote seed germination (Teng et al., 2010) and increase seed number (Deng et al., 2025) by reducing the content of ABA. Additionally, seed size and vegetative growth duration can affect plant size and yield (Gómez-Fernández and Milla, 2022). Therefore, the ‘fast-growing small-fruit’ phenotype in *ZjGC*-overexpressing tomatoes may mainly result from smaller seeds and a shorter vegetative growth period.

Moreover, by adding exogenous cGMP to the culture medium of jujube calli, we found that not only the endogenous cGMP content but also the endogenous GA3 content were significantly increased. Combined with the anatomical observation, it was more intuitive to see that the number of cells in the jujube calli added with cGMP significantly increased compared to the CK. Our experiment also observed that with the increase of cultivation days, the structure of callus tissue gradually became hard and tight, presenting a type I callus morphology, which is easy to differentiate into buds through organ pathways. Therefore, we speculated that cGMP may also accelerate the cell division and differentiation of callus.

## Conclusion

This study discovered the first *GC* gene in woody plant jujube (ZjGC), and confirmed that the ZjGC can catalyze GTP to cGMP by *in vitro* enzymatic property analysis, transient overexpression in jujube fruit *in vivo*, and generation of transgenic plants of Micro-Tom tomato. Overexpressing *ZjGC* in tomato showed that *ZjGC* has vital biological functions of promoting seed germination, shortening juvenile period, accelerating fruit development, increasing the number of seeds, and decreasing the size of fruit and seed. It was discovered for the first time that there is a feedback regulation mechanism of cGMP to hormones in plant. The addition of exogenous cGMP to jujube calli significantly increased the concentrations of endogenous cGMP and GA3, resulting in a considerable increase in calli growth. Furthermore, exogenous cGMP treatment can influence cell division and differentiation. This study lays a solid foundation for further study and utilization of GC and cGMP as well as the research on phytohormone signaling in plants.

## Materials and methods

### Materials

We extracted RNA from ‘Dongzao’, a leading variety of Chinese jujube whose genome fully sequenced and assembled by our group (Liu et al., 2014; Yang et al., 2023), and used it as a template to clone candidate GC genes. White-ripe stage fruits were used for transient overexpression analysis. The wild type Micro-Tom was obtained from Biorun Biotechnology Company (Wuhan, China), and the Micro-Tom genetic transformation system was used to obtain transgenic tomato plants. Jujube calli were cultured at the Research Center of Chinese Jujube, Hebei Agricultural University, China, and used for exogenous cGMP treatment.

### RNA isolation

Total RNA was isolated directly from jujube fruit and tomato tissues or organs using the RNA prep Pure Plant Plus Kit (TIANGEN Biotech Beijing Co., Ltd.) according to the manufacturer’s protocol. The *ZjGC* gene was amplified and cloned using “Dongzao” as a template.

### Amplification and cloning of *ZjGC* from *Z. jujuba* Mill.

(1) Identification of the complete coding sequence (CDS) of *ZjGC*. The partial *GC* gene sequence of *Arabidopsis thaliana* was used as the query condition to search *ZjGC* from jujube (Ludidi and Chris, 2003). (2) The amplification of the cDNA sequence of the *ZjGC* fragment. The *ZjGC* fragment cDNA sequence was obtained from jujube genome using homologous cloning. Primer pairs were designed using Primer Premier 5.0 software. (forward: 5 ‘ATGTGGCCTTTATATATCCTTTTCA - 3, the reverse: 5’ - CTAAGCAGAAAGCTGTAGAGAATTG - 3, N is degenerate base), according to the conserved nucleotide sequences of putative cloned GCs in the NCBI database (National Center for Biotechnology Information). The PCR products were ligated into the pMD19-T vector Cloning Kit (Takara) and sequenced. (3) To confirm the CDS of *ZjGC*, PCR amplification was performed with specific primers designed according to the predicted *ZjGC* CDS. The primer pairs were as follows: 5′- TACAGCATTCAGTCTTTGGC-3′ (forward) and 5′- GAATAGCGTCTCCACTCGTA-3′ (reverse). The PCR product ligation and sequencing were the same as those described above.

### Phylogenetic analysis of GC

Phylogenetic analysis of the catalytic domains of GCs. The ML (Maximum likelihood) tree was constructed from the amino acid sequences of ZjGC using MEGA 5.2 with 1000 bootstrap replicates, using the online website (https://www.chiplot.online/) to render the color and shape of evolutionary trees (Xie et al., 2023).

### *Agrobacterium tumefaciens* preparations

The complete coding sequences of *ZjGC* genes were ligated into the green fluorescent protein (GFP) vector pCambia1302 with 35S promoter (GENCEFE Biotech Wuxi, China), and transferred into *Agrobacterium tumefaciens* strains GV3101 (Keylab Biotech, Jiangsu, China) by the freeze-thaw method. After that screened for positive transformants in LB agar plate supplemented with kanamycin (100 μg/mL) and rifampicin (100 μg/mL). Single colonies were picked and incubated into 20 mL LB medium at 28 °C, 200 rpm, then detected by PCR and harvested at an OD600 of 1.5 to 2. A*. tumefaciens* was resuspended for transient overexpression of *ZjGC* in jujube fruit *in vivo*. At the same time, PCR-positive colonies were inoculated into a 20 mL LB medium containing 100 μM acetosyringone (Sigma-Aldrich) at 28 °C, with shaking at 200 rpm. After centrifugation, the bacteria were re-suspended at an OD600 of 0.6 to 0.8 with MS liquid medium for the transformation of cotyledons (Wang et al., 2024).

### Transient overexpression of *ZjGC* in jujube fruit *in vivo*

Three independent *Z. jujuba* “Dongzao” trees cultivated at the ZanHuang jujube Experiment Station, were used for transient overexpression analysis. Each fresh white mature fruit was injected with 2 mL of *A. tumefaciens* strain GV3101 solution containing 35S::*ZjGC-GFP* using a syringe. 35S::GFP was injected as a negative control. After infiltration, the fruits were covered with aluminum foil for 24 h before removal. Samples were collected before injection (0 h), 24 h, 48 h, and 72 h after injection, when collecting, it is necessary to select fruits with consistent growth, each treatment required 5 fruits to be collected from each tree. Samples then were ground in liquid nitrogen for analysis of *ZjGC* expression level and cGMP content.

### Gene construction, protein expression and purification

CDSs of *ZjGC* were synthesized by Anhui General Biological Systems Co., LTD and inserted into the pET15a vector. The construct was transferred to BL21 (DE3) *E. coli* receptor cells (TIANGEN Biotech, Beijing, China), and grown at 36 °C in LB medium with 1 mM isopropyl-1-thio-β-D-galactoside (IPTG) for protein induction. Proteins were purified with nickel-nitrogen triacetic acid resin according to the manufacturer’s protocol, and then the appropriate amount of sample was to determine protein purity by SDS-polyacrylamide gel electrophoresis (Yuan et al., 2022).

### *In vitro* guanylate cyclase activity of candidate GC proteins

The activity of GC proteins was determined by monitoring the release of cGMP from GTP. All reactions were carried out in a 500 μL mixture, recombinant protein was incubated for 20 min at 30 °C in 50 mM Tris−HCl (pH 7.5) with 0.5 mM GTP concentrations, 10 mM divalent cation (Mn^2+^, Mg^2+^, Ca^2+^) cofactor and 20 μg purified protein. Generation of cGMP was determined by ELISA Kit (LE-Y1602, Hefei Lai Er Bio-Tech Co., Ltd).

### Overexpression of *ZjGC* in Micro-Tom tomato

Transgenic Micro-Tom plants overexpressing *ZjGC* genes were regenerated from leaf disks after *Agrobacterium tumefaciens*-mediated transformation. The seeds were surface-sterilized by immersion for 30 s in 70% (v/v) ethanol followed by 15 min in 5% (v/v) NaClO and 5 rinses with sterile distilled water. Sterilized seeds were cultured on 0.5 × MS medium, when the cotyledons expanded fully, cut the cotyledons and placed on MS + 1 mg/L zeatin (ZT) for a 48 h preculture. Then, the cotyledon explants were subjected to *Agrobacterium tumefaciens* GV3101 infection solution for 15 min and transferred to MS + 2 mg/L ZT medium for a 48 h co-cultivation. After transformation, the cotyledon explants were put on screening media (MS + 2 mg/L ZT + 10 mg/L hygromycin + 300 mg/L cefotaxime) for regeneration. When calli with shoot buds formed from the cotyledon fragment, the cotyledons were cut off and transferred to rooting medium, MS + 1 mg/L IAA (Indole acetic acid) + 10 mg/L hygromycin + 300 mg/L cefotaxime. Finally, rooted plants were transferred to soil and grown to maturity. The Micro-Tom plants that regenerated from untransformed cotyle-dons were taken as control. The regenerated transgenic plants confirmed by PCR were planted in the greenhouse. Only the homologous T2 lines were used for phenotyping, Tomato RNA was extracted using RNA prep Pure Plant Plus Kit. All fresh plant samples were ground in liquid nitrogen completely and stored at -80 °C for further use.

### Effect of exogenous cGMP on calli of jujube

The jujube seed-induced calli were selected as the material for the calli test. The subculture medium of calli was MS 4.23 g/L, AGAR 5.5 g/L, maltose 20 g/L, trace elements (M2, M3, M4, M5), and the pH value was adjusted by NaOH (5.8). Plant growth regulators: naphthalene acetic acid (NAA), thidiazuron (TDZ). The culture medium was treated with different concentrations of cGMP (0, 50,100,200,300 μM) (Ma, 2007; Jia and Ma, 2020), and the calli was cultured in Petri dishes. For each group, three dishes were set up and repeated three times. The materials were incubated at 25 °C for 10 d and 20 d. Before sampling, the morphological changes of calli were observed and photographed. All samples were carefully picked up with tweezers and put into new petri dishes, weighed and recorded. Rinsed briefly with small water flow, and used dust-free paper to repeatedly absorb the water, then frozen in liquid nitrogen and powdered.

### Determination of cGMP content

To measure cGMP in plant samples, 1 g of fresh samples were ground in liquid nitrogen completely, and mixed with 5 mL of water. The mixture was subjected to a 60 °C water bath for 3h, after centrifugation at 12000 rpm for 10 min, the supernatants were filtered through a 0.22 μm filter and tested. The cGMP content was determined by HPLC (Agilent 1200 Series) (Liu et al., 2023).

### Determination of phytohormone contents

In the experiment, methanol was used as the extraction agent to prepare a standard mixture, the mixed liquids included IAA (Indoleacetic acid), ABA (Abscisic acid), GA3 (Gibberellic acid 3), GA4 (Gibberellic acid 4) and ZT (Zeatin). A total of 0.5 g frozen plant samples were ground to powder in liquid nitrogen, suspended in a 3 mL methanol mixture (Methanol/water/formic acid, 15/4/1), and extracted at 4 °C for 16 h. After 30 min of low-temperature ultrasound oscillation, centrifuged for 5 min at 4 °C, 12000 rpm, following the supernatants were transferred and concentrated in new centrifuge tubes. Then, the concentrated liquids were dissolved in 1 mL 80% methanol and passed through a 0.22 μm filter for LC - QQQ (Agilent1290-6495C) analysis (Gong et al., 2016).

### Micro-Tom tomato model

The material of the tomato model was provided by Figdraw 2.0.

### Analysis of *ZjGC* promoter

*ZjGC* promoter was analyzed online by the PlantPAN 4.0 website (https://plantpan.itps.ncku.edu.tw/plantpan4/index.html) and visualized using ChiPlot (https://www.chiplot.online/).

### Statistical analysis

The data were collated using Excel software and the mean was used to represent the overall level of each trait in the sample. One-way ANOVA was used for statistical evaluations by SPSS Statistics 26.0 software. Graph Pad Prism 10 was used for Charting.

## Data Availability

The datasets presented in this study can be found in online repositories. The names of the repository/repositories and accession number(s) can be found in the article/supplementary material.
